# Complications and complaints in craniofacial fractures – Finnish national data for 20 years

**DOI:** 10.2340/aos.v83.40570

**Published:** 2024-05-28

**Authors:** Inka Luotamo, Johanna Snäll, Miika Toivari

**Affiliations:** a Department of Oral and Maxillofacial Diseases, Kymenlaakso Central Hospital, Kotka, Finland; b University of Helsinki, Helsinki, Finland; c Department of Oral and Maxillofacial Diseases, University of Helsinki, Helsinki, Finland; d Helsinki University Hospital, Helsinki, Finland

**Keywords:** Craniofacial fracture, complaint, malpractice, delayed diagnosis, permanent harm

## Abstract

**Objective:**

Research on reasons for malpractice claims in oral and maxillofacial surgery is scarce. The aim of this study was to investigate the causes and prevalence of permanent harm among craniofacial fracture related malpractice claims.

**Materials and methods:**

A retrospective register study was designed and implemented. All patients with a complaint and a diagnosis of facial or cranial fracture were included. The main outcome was the presence of permanent harm, and the predictor variable was the cause of complaint. Chi-square test was used for estimation of statistical significance.

**Results:**

Delay in correct diagnosis was the leading cause of malpractice claims (63.2%), and permanent harm was found in 23.1% of the population. 82.4% of injuries were facial fractures in total population. 65.3% (*n* = 98) of facial trauma were related with delayed diagnostics (*p <* 0.001). Permanent harm was more frequent in patients with delayed diagnosis (71.4%) than those without (60.7%, *p* = 0.299).

**Conclusions:**

Claims of craniofacial trauma are related with under-diagnostics, and un-diagnosed facial fracture can lead to a high rate of permanent harm. Systematic clinical evaluation and facial trauma specialist consultation is recommended to set early correct diagnosis for and improve treatment of craniofacial trauma patients.

## Introduction

The risk of complications and complaints is always present in medical treatment and care. A recent review concluded that 49–65% of complaints are related to general practice. However, surgery has been reported among the most high-risk specialties receiving complaints [1, 2], the risk being up to 12-fold higher than in general practice [3].

The factors which have been associated with malpractice complaints in general surgery have been history of previous claims, lack of diagnosis, heavy workload, practitioners’ older age, monitoring, and follow-up. [1, 3, 4] In addition to the factors previously mentioned, communication between practitioner and patient has a clear impact on dissatisfaction with treatment [4].

In maxillofacial surgery, malpractice complaints have been reported in relation to orthognathic surgery, facial esthetics, temporomandibular joint disorder, dental implantology and tooth extraction related complications, facial injury related sensory disturbances, and scarring [5, 6–9]. Malocclusion, scar contracture, diplopia, malunion, wound dehiscence, and infection are well-documented complications in facial trauma [10]. A recent meta-analysis revealed that mandibular fracture infections (66.7%), followed by excessive scarring (20.7%), wound dehiscence (13.8%), and insufficient reposition (13.8%) were the most common types of complications in facial fracture patients [11].

Publications focussing on the background of demographics in facial trauma malpractice and complications are a few [12, 13]. The purpose of this study was to investigate the demographics and causes leading to malpractice complaints in craniofacial fracture patients in Finland. The specific aim was to clarify the reasons for complaints and the prevalence of permanent harm. Our hypothesis was that fracture treatment-related complications are the leading group in major craniofacial injuries.

## Materials and methods

### Study design and sample description

Patient liability insurance is statutory for all companies, institutions, private entrepreneurs, and healthcare professionals engaged in health and medical care activities in Finland. The insurance centre determines whether the claim is to be compensated and whether permanent harm has occurred, and corrective instructions are given in the case of malpractice. The records of all patients with a closed complaint of facial, cranial, or craniofacial fracture treatment were reviewed from the Finnish Patient Insurance Centre from 1 January 1999 to 31 December 2019. Complaints without a definitive decision were excluded from the study.

### Study variables

All patients with a diagnosed facial, cranial, or combined craniofacial fracture were included in the study. The main outcome was the presence of permanent harm (i.e., present or absent).

The primary predictor variables comprised the cause of complaint grouped as follows: (1) delay of correct diagnosis or treatment with or without other complaints, (2) complication of fracture treatment or end-result of treatment, and (3) other. In addition, the number of the following specific causes of complaints was presented: delay of correct diagnosis or treatment, complication of fracture or end-result of treatment, under-diagnostics of associated injury (AI), improper instructions or medical treatment, and impolite or unprofessional behaviour.

The explanatory variables were sex, age, mechanism of injury, type of fracture, mortality due to injuries (i.e., yes or no), and missed diagnosis of AI. The injury mechanism was classified as follows: (1) fall on the ground, (2) assault, (3) hit by blunt object, (4) bicycle accident, (5) fall from height, (6) motor vehicle accident (MVA), or (7) other. The type of fracture was classified as follows: (1) cranial, (2) facial, or (3) craniofacial.

### Data analysis

Descriptive statistics were calculated for all variables. A Chi-square test was used to evaluate the statistical significance between groups.

### Ethical approval

The internal review board of the patient liability insurance centre of Finland approved the study protocol (PVK16042019).

## Results

Table 1 shows the descriptive statistics of 182 patients with a malpractice committee complaint. Of these patients, 53.8% were male, the average age being 38 years. The most common cause of trauma was falling on the ground (34.1%), followed by assault (24.7%). Most injuries were pure facial fractures (82.4%). Delay of correct diagnosis or medical treatment was the predominant cause of complaint (63.2%). Permanent harm was determined for 23.1% of the patients, and 71.4% (130 patients) had financial coverage for their complaint.

**Table 1:** Descriptive statistics of 182 patients with malpractice committee complaint.

Study variables	*n* = 182	% of *n*
**Sex**		
Male	96	53.8
Female	86	47.3
**Age (in yrs.)**		
Range	0–91	
Mean	38.0	
**Age group (in yrs.)**		
<18	34	18.7
19–39	60	33.0
40–59	68	37.4
60+	20	11.0
**Mechanism**		
Fall on the ground	62	34.1
Assault	45	24.7
Hit by object	20	11.0
Bicycle accident	18	9.9
Fall from height	17	9.3
MVA	17	9.3
Other	3	1.6
**Type of fracture**		
Facial	150	82.4
Cranial	19	10.4
Cranio-facial	13	7.1
**Number of complaint causes**		
Single cause	138	75.8
Two causes	36	19.8
Three or more causes	5	2.7
Cause of complaint remains unknown	3	1.6
**Cause of complaint**		
Delay of correct diagnosis or treatment ± end result ± complication of fracture treatment	115	63.2
Complication of fracture or end result of treatment	48	26.4
Improper instructions or medical treatment	10	5.5
Impolite or un-professional behaviour	5	2.7
Under diagnosis of AI	1	0.5
Unknown	3	1.6
**Permanent harm**		
Yes	42	23.1
**Missed AI present**		
Yes	10	5.5
**Mortality**		
Yes	4	2.2
**Compensation paid**		
Yes with or without permanent harm	90	49.5
Yes with permanent harm	40	22.0

AI: associated injury; MVA: motor vehicle accident; yrs.: years.

The associations between sex, age, mechanism, type of fracture, mortality, missed AI, and cause of complaint are presented in Table 2. The frequency of 40- to 50-year-old patients was highest in three groups with different causes of complaint, 35.7% in delay related causes, 39.6% in complication of treatment, and 42.1% in other group, (*p* 0.574). Falling on the ground (37.4%) was the most common cause of injury in the group complaining of delayed diagnosis or treatment, and assault (39.6%) in the group citing complication of fracture treatment or end-result of fracture treatment, the difference being significant (*p* = 0.026). Facial fracture was by far the most common type of injury in the delay (85.2%), complication (87.5%), and other (52.6%) groups, the difference being significant (*p* < 0.001). Altogether 7 of the 9 missed AIs were associated with complaints of delayed diagnosis or treatment; the association was however, not significant (*p* = 0.886).

**Table 2:** The association between sex, age, type of fracture, mortality, missed AI, and cause of complaint.

Study variables	Delay of correct diagnosis or treatment ± end result ± complication of fracture treatment		Complication of fracture or end result of treatment		Other	%	*P* *
	*n* = 115	%	*n* = 48	%	*n* = 19		
**Sex**							0.839
Male	61	53.0	24	50.0	11	57.9	
Female	54	47.0	24	50.0	8	42.1	
**Age (yrs.)**		0.0					
Range	0–91		6–86		11–75		
Mean	36.84		39.35		44.63		
**Age group (yrs.)**							0.574
<18	25	21.7	8	16.7	1	5.3	
19–39	38	33.0	16	33.3	6	31.6	
40–59	41	35.7	19	39.6	8	42.1	
60+	11	9.6	5	10.4	4	21.1	
**Mechanism**							0.026
Fall on the ground	43	37.3	13	27.1	6	31.6	
Assault	25	21.7	19	39.6	1	5.3	
Hit by object	13	11.3	3	6.3	4	21.2	
Bicycle accident	14	12.2	3	6.3	5	26.3	
Fall from height	10	8.7	2	4.2	1	5.3	
MVA	8	7.0	8	16.7	1	5.3	
Other	2	1.7	0		1	5.3	
**Type of fracture**							<0.001
Facial	98	85.2	42	87.5	10	52.6	
Cranial	11	9.6	1	2.1	7	36.8	
Cranio-facial	6	5.2	5	10.4	2	10.5	
**Mortality**							0.027
Yes	2	1.7	0		2	10.5	
No	113	98.3	48	100.0	17	89.5	
**Missed AI**							0.886
Yes	7	6.1	2	4.2	1	5.3	
No	108	93.9	46	95.8	18	94.7	

yrs.: years;

* : chi-square; other: under diagnosis of AI, improper instruction or medical treatment, impolite or un-professional behaviour; AI: associated injury; MVA: motor vehicle accident.

Table 3 presents the associations between sex, age, mechanism, type of fracture, mortality, missed AI, and main outcome. Statistical significance did not emerge between any of these variables. However, the proportion of men was higher in patients with permanent harm (59.5%) than in the control group (50.7%). Permanent harm was clearly more frequent in patients aged 40–59 years than in any other age group. Assault (35.7%) and fall on ground (26.2%) were the most common cause of trauma in patients with permanent harm, and their injuries were mainly (90.5%) facial fractures. The rates of mortality (*n* = 2) and missed AI (*n* = 2) were even between the two groups.

**Table 3:** The association between sex, age, mechanism, type of fracture, mortality, missed AI, and main outcome.

Study variables	Permanent harm	%	Permanent harm	%	*P* *
	Present		Absent		
	*n* = 42		*n* = 140		
**Sex**					0.316
Male	25	59.5	71	50.7	
Female	17	40.5	69	49.3	
**Age (yrs.)**					
Range	11–75		0–91		
Mean	39.64		37.94		
**Age group (yrs.)**					0.302
<18	7	16.7	27	19.3	
19–39	13	31.0	47	33.6	
40–59	20	47.6	48	34.3	
60+	2	4.8	18	12.9	
**Mechanism**					0.446
Fall on the ground	11	26.2	51	36.4	
Assault	15	35.7	30	21.4	
Hit by object	6	14.3	14	10.0	
Bicycle accident	3	7.1	15	10.7	
Fall from height	3	7.1	14	10.0	
MVA	4	9.5	13	9.3	
Other	0		3	2.1	
**Type of fracture**					0.148
Facial	38	90.5	112	80.0	
Cranial	1	2.4	18	12.9	
Cranio-facial	3	7.1	10	7.1	
**Mortality**					0.196
Yes	2	4.8	2	1.4	
No	40	95.2	138	98.6	
**Missed AI**					0.812
Yes	2	4.8	8	5.7	
No	40	95.2	132	94.3	

* : chi-square; AI: associated injury; yrs.: years; MVA: motor vehicle accident.

Table 4 shows the association between the cause of complaint and the presence of permanent harm. Delayed diagnosis or treatment (71.4%) was by far the most common cause of complaint in patients with permanent harm; however, their rate did not differ significantly from patients without permanent harm (60.7%, *p* = 0.299).

**Table 4:** The association between the cause of complaint, and presence of permanent harm.

Study variables	Permanent harm	%	Permanent harm	%	*P* *
	Present		Absent		
	*n* = 42		*n* = 140		
**Cause of complaint**					
Delay of correct diagnosis or treatment ± end result ± complication of fracture treatment	30	26.1	85	73.9	0.299
Complication of fracture or end result of treatment	10	20.8	38	79.2	
Other	2	10.5	17	89.5	

* : chi-square.

**Figure 1:**
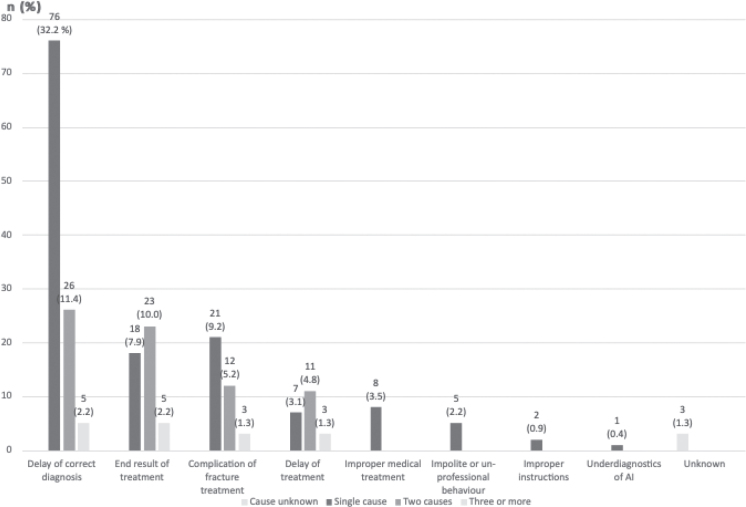
Number of individual causes of malpractice complaints.

The frequency of solitary causes of complaint is presented in Figure 1. Delay of correct diagnosis was by far the most common cause of complaint (*n* = 107), followed by the end-result of treatment (*n* = 46), and complications of fracture treatment (*n* = 36). The cause of complaint could not be determined for three patients.

## Discussion

Publications focussing on facial and cranial trauma malpractice and complications are few. The purpose of this study was to investigate the demographics and causes leading to malpractice complaint in craniofacial fracture patients in Finland. We specifically aimed to clarify the reasons for complaints and whether permanent harm had occurred. The hypothesis was that fracture treatment-related complications are the leading group in major craniofacial injuries.

The hypothesis was rejected. The predominant cause of complaint in craniofacial fracture patients was delayed diagnosis or treatment with or without other complaint causes (63.2%). Delay-related complaints were more frequent in patients with permanent harm (71.4%) than in those without (60.7%), although the difference was not significant. Thus, in addition to the prevention of surgical complications, there is a marked need in the field of facial traumatology to improve the early diagnosis and initial care of these patients.

Fracture limited to the facial area was by far the most common fracture type (82.4%). Interestingly, regarding injury mechanisms, fall on ground (34.1%) and assault (24.7%) differed regarding the cause of complaint. Patients who had been assaulted complained more often of fracture complications and/or end-results, whereas patients sustaining ground level falls complained of delayed diagnosis and/or treatment (*p* < 0.026).

An injury or its treatment can negatively impact a patient’s social encounters, mental health, and ability to work, thus lowering their quality of life [14–17]. Ji et al. [18] concluded that up to 34.6% of all patients undergoing a maxillofacial procedure sustain at least minor permanent harm, with the rate of death being up to 5.3%. Our study revealed that 23.1% of craniofacial fracture patients suffered permanent harm, 4.8% of these patients having undiagnosed AI and 2.2% dying from their injuries. Thus, the rates of malpractice complaints of craniofacial fracture patients are clearly lower than those of maxillofacial surgery patients in general.

Permanent harm was most typically found in middle-aged (47.6%) males (59.5%) sustaining their injuries from interpersonal violence (35.7%). In the literature, a fracture of the zygomatico-orbital complex has been the most typical facial fracture type in these patients. The occurrence of this fracture type was high in the 1980s and the 1990s (42.0% for both) [19], falling at the beginning of the 21st century (38.3%) [20]. The complications reported in the literature, for example, in orbital blow-out fractures have been diplopia, infraorbital nerve injury, and esthetics which are fortunately often transient but affect the quality of life severely [21]. One of the factors proven to diminish long-lasting neurosensory disturbance has been short delay (<1 week) to surgical intervention when surgery is clinically indicated [22].

Indeed, the current study revealed that a delay-related cause was by far the most common cause of malpractice complaint (63.2%), compared with other causes. The result of previous publications on facial trauma complaints (1.9–34.8%) were clearly different [13, 18]. One explanation for the discrepancy may be different legal processes; for example, the study by Ji et al. examined material including claims settled before court and at court [18], whereas the Finnish malpractice complaint process is an assisted process with a low contact threshold, not court-related, and even the suspicion of a treatment-related harm is evaluated. The diagnostic delay can have contributory factors such as trauma scene, geographical location, influence of alcohol or other intoxicant substances, and arrestment which can affect to the time span from injury to correct diagnosis and treatment path [23, 24]. In addition to the previous, literature has shown that 8.8% of medical mistakes are related to the event of negligence [7], which should not be involved in medical care.

Under-diagnostics and under-triage are well-known problems. For example, the rate of under-diagnostics of geriatric general trauma varies from 15.0 to 69.1% under the primary trauma triage [25–28]. In relation to facial fracture diagnostics, Kannari et al. [29] stated that up to 20.1% of patients at least 60 years of age do not receive correct fracture diagnosis under primary evaluation after the injury. In the present study, 63.1% of patients with a mean age of 36.81 years made a complaint related to diagnostic delay. Ugboko [23] reported a complication rate of 22.9% combined with a primary contact within the first 7-day period from injury for 83.4% of the study population, whereas the more recent results of Stanford-Moore [24] reveal that 81.0% of complications were found after a delay of 3 days from injury. The delay in diagnosis can lead to functional deficiencies, increased risk of infection, malunion, and unnecessary pain [23]. Proper education for facial injury diagnostics, meticulous repeated status, and low-threshold facial traumatologist consultation are recommended to avoid under-diagnostics of facial fractures in middle-aged patients also.

The main strength of this study was coverage of both private and public sector complaints over a 20-year period in Finland. Our study highlights fracture diagnostics as the primary cause of complaints.

A limitation of the study is its retrospective nature: Firstly, a prospective study would have given more detailed information on solitary complications. Secondly, the study compares the malpractice claims to the site of facial trauma (upper, middle, and lower facial third), whereas the specific type of facial fracture would have given even more information.

In conclusion, our hypothesis of fracture treatment-related complaints being the leading cause of malpractice claims was rejected. Low-threshold facial traumatologist consultation is recommended to establish appropriate diagnosis of facial fracture, to prevent harmful delay in diagnostics and fracture treatment, and to avoid under-diagnostics and permanent harm also in middle-aged men suspected of having a craniofacial fracture.

## Disclosure statement

The authors have no potential conflicts of interest to report.
